# Association between novel adipocytokines adiponectin, vaspin, visfatin, and thyroid: An experimental and clinical update

**DOI:** 10.1530/EC-13-0061

**Published:** 2013-11-18

**Authors:** Nese Cinar, Alper Gurlek

**Affiliations:** 1 Department of Endocrinology and MetabolismHacettepe University School of Medicine06100, Sihhiye, AnkaraTurkey

**Keywords:** thyroid, metabolism

## Abstract

Adipose tissue secretes a variety of active biological substances, called adipocytokines, that act in an autocrine, paracrine, and endocrine manner. They have roles in appetite control, thermogenesis, and thyroid and reproductive functions. All these molecules may lead to local and generalized inflammation, mediating obesity-associated vascular disorders including hypertension, diabetes, atherosclerosis, and insulin resistance. Thyroid dysfunction is associated with changes in body weight, thermogenesis, and energy expenditure. The connections between cardiovascular risk factors such as dyslipidemia, impaired glucose tolerance, insulin resistance, atherosclerosis, and thyroid dysfunction have been reported in several studies. The adipocytokines serve as causative or protective factors in the development of these disorders in the states of thyroid dysfunction. Abnormal levels of adipocytokines (adiponectin (ADP), leptin, resistin, vaspin, and visfatin) in hypo- and hyperthyroidism have been reported with controversial results. This review aims to update the implication of novel adipokines ADP, vaspin, and visfatin in thyroid dysfunction.

## Introduction

Adipose tissue is a complex organ including adipocytes, immune cells, fibroblasts, blood vessels, and collagen fibers. It is classified as brown adipose tissue (BAT) and white adipose tissue (WAT). WAT is the predominant type because BAT regresses after birth. WAT serves as a storage site for energy in the form of triglycerides. Over the past decade, it has been recognized that adipose tissue has important functions other than energy storage, such as secreting a variety of endocrine, paracrine, and autocrine hormones; cytokines; and growth factors which influence local adipose tissue and different organs/tissues. These include CNS, liver, pancreas, and the skeletal muscles. Leptin, adiponectin (ADP), vaspin, visfatin, interleukin 6 (IL6), plasminogen activator inhibitor type 1, and resistin are among the adipocytokines secreted from the WAT. In obese humans, these adipocytokines play a role in the development of a cluster of metabolic abnormalities characterized by central obesity, dyslipidemia, type 2 diabetes, hypertension, and cardiovascular complications, by promoting a low-grade WAT inflammation (1, 2).

Thyroid hormones are involved in the regulation of body metabolism. Their effects include the stimulation of resting metabolic rate, increase in energy expenditure, modulation of responsiveness to catecholamines, and thermogenesis in adipose tissue (3, 4). Disturbances in thyroid function lead to changes in body weight, muscle mass, and fat tissue. Thyroid-stimulating hormone (TSH) receptors have been found in the adipose tissues, indicating that they play a role in the regulation of the adipocytokines which are involved in the regulation of energy balance (5). This article focuses on the novel adipocytokines, namely ADP, vaspin, and visfatin in the context of thyroid dysfunction and the associated changes in adipose tissue and insulin resistance. The reason for choosing them is the increasing evidence regarding their changes in states of thyroid dysfunction.

## Adiponectin

ADP is a 244 amino acid protein that is the most abundant gene product of the adipose tissue, gene transcript 1 (apM1) (6). It accounts for 0.01% of total plasma proteins with plasma concentrations ranging from 5 to 30 mg/ml (7). Two different types of ADP receptors (AdipoR), 1 and 2, have been identified (8). AdipoR1 is expressed primarily in the muscle and AdipoR2 is expressed primarily in the liver. Binding of ADP to AdipoR1 and AdipoR2 results in increased glucose uptake and fatty acid oxidation in the skeletal muscle (AdipoR1) and decreased glucose output from the liver (AdipoR2) (9). ADP circulates in three forms, trimer, hexamer (also called low molecular weight oligomer), and high molecular weight (HMW) multimers. The biological effects of ADP depend on plasma concentrations, properties of different ADP isoforms, and tissue-specific expression of the ADP receptor subtypes. The most active form of ADP is the HMW (10).

ADP has anti-atherogenic, anti-diabetic, and anti-inflammatory properties. Obese patients have significantly lower ADP plasma levels than lean subjects (7), and a strong and consistent negative correlation exists between ADP levels and insulin resistance (11, 12). Low levels of ADP are associated with several diseases such as atherosclerosis, type 2 diabetes, abdominal obesity, hypertriglyceridemia, low HDL, hypertension, and metabolic syndrome (13). On the other hand, weight loss or insulin-sensitizing agents lead to increases in ADP levels in association with improved insulin resistance (11, 12).

ADP plays a role in the regulation of body temperature and basal metabolic rate. ADP has structural similarities with hibernation-associated plasma proteins HP-27, HP-25, and HP-20 in chipmunks, suggesting a role for it in adaptive thermogenesis (14). ADP may increase thyroid hormone synthesis, especially free thyroxine (fT_4_), as a result of the C-terminal globular structure of ADP interacting with the gC1q receptor found in the mitochondria of thyroid cells (15). Given that thyroid hormones share some physiological actions with ADP (i.e. such as reduction of body fat by increased thermogenesis and lipid oxidation) (16), it is conceivable that ADP may interact with thyroid axis. Thyroid hormones are associated with insulin resistance (17), and a relationship between ADP and thyroid hormones may exist via either direct or indirect interactions between them.

Controversial results are reported for the experimental studies on hypo/hyperthyroid animals, concerning the association between ADP levels and thyroid hormones. Hypothyroid rats have either increased or unchanged serum ADP levels, while unchanged or increased serum ADP levels are found in hyperthyroid rats (18, 19). This might be due to the extent of thyroid hormone alterations in each study. The levels of ADP mRNA in the adipose tissue are decreased in hypothyroid rats compared with controls, and this decrease is in parallel with the decrease in triiodothyronine (T_3_), T_4_, fT_3_, and fT_4_ concentrations (20). In hyperthyroid rats, adipose ADP expression is increased in parallel with an increase in thyroid hormones, the opposite is observed in hypothyroid rats (20). On the other hand, Kokkinos *et al*. (18) demonstrated increased ADP levels in hypothyroid rats, whereas no significant change was observed in the ADP levels after the administration of thyroid hormone. Cabanelas *et al*. (21) showed that T_3_ administration in rats had no significant effect on ADP secretion in visceral (epididymal) and subcutaneous (inguinal) adipose tissues, while ADP mRNA expression was downregulated by T_3_ in the subcutaneous adipose tissue, but not in the visceral adipose tissue (VAT). The authors suggest that ADP mRNA response to T_3_ changes depending on type and anatomical site of the WAT. In contrast, T_3_ administration was shown to increase ADP mRNA expression and release in a culture of mouse brown adipocytes (22). Seifi *et al*. (23) demonstrated that methimazole administration resulted in a decrease in mRNA levels of adipoR1 and adipoR2 in WAT in hypothyroid rats, whereas mRNA levels of these ADP receptors are increased in the hyperthyroid group. ADP receptor gene expression levels in WAT had positive correlations with thyroid hormone concentrations, suggesting that *AdipoR1* and *AdipoR2* gene expression are regulated by thyroid hormones in hypo- and hyperthyroidism.

In humans, the results of studies of the interaction between thyroid hormones and ADP are conflicting. There are few studies to date regarding the changes in the release of ADP in thyroid dysfunction. The results and essential information for these studies are summarized in Table 1.

In the studies mentioned earlier, the effect of thyroid hormones on the ADP levels revealed contrary results and no definite conclusion can be drawn. This might be due to differences in patients' characteristics, degree and duration of thyroid hormone dysfunction, metabolic effects of other hormones, and possible effects of intermediate metabolism. Differences in the duration of the studies and small sample size included in the studies may be other possible causes of this inconsistency. Moreover, the presence of autoimmunity in thyroid disorders may play a role in this controversy, as an association between thyroid antibodies and ADP was shown in the studies.

Thyroid hormones are important regulators of lipid and carbohydrate metabolism. Thyroid dysfunction leads to dyslipidemia and impairment of glucose metabolism (43). The underlying mechanisms of these disturbances are still unknown. AdipoR1 and AdipoR2 were reported to play a role in glucose and lipid metabolism. The disruption of these receptors in the mouse liver results in increase in tissue triglyceride content, inflammation, and oxidative stress leading to insulin resistance and marked glucose intolerance (44). *ADP* (*ADIPOQ*) gene expression is negatively correlated with LDL-C and triglyceride levels in hypothyroid rats and positively correlated with glucose and HDL-C levels in hyperthyroid rats. Taken together, thyroid hormones may modulate lipid and carbohydrate metabolism via changes in ADP receptor expression in the adipose tissue. Hypothyroidism is associated with atherosclerosis, dyslipidemia, diastolic hypertension, impaired endothelial dysfunction, and insulin resistance (45). ADP precludes the development of atherosclerotic plaques in the injured vessel wall, which was reported in a recent study (46), which might indicate that decreased ADP expression in hypothyroidism has a role in the pathophysiology of atherosclerosis in these patients. The biological reason for increased plasma ADP levels in the adipose tissue in hyperthyroidism is unclear. Thyrotoxicosis is associated with reduction of fat and muscle mass and depletion in lipid storage. Insulin resistance in the liver and peripheral tissues is observed in hyperthyroid patients. Therefore, increased ADP levels might represent a compensatory mechanism against the insulin resistance observed in the hyperthyroid state. Further studies are needed to confirm all these hypotheses.

## Vaspin

VAT-derived serine protease inhibitor (Vaspin) is a novel insulin-sensitizing adipocytokine identified from VAT of obese diabetic Otsuka Long-Evans Tokushima Fatty (OLETF) rats (47). Hida *et al*. (47) reported that administration of vaspin to OLETF rats results in improvement in glucose tolerance and insulin sensitivity. Recently, vaspin single-nucleotide polymorphism rs2236242 has been found to be positively associated with type 2 diabetes in 2759 participants in the KORA F3 study (48). The reported AA genotype (A represents the minor allele sequence) bears an increased risk of diabetes independent of obesity, suggesting a link between vaspin and glucose metabolism. El-Mesallamy *et al*. (49) found higher vaspin concentrations in both obese and non-obese type 2 diabetes patients than in controls, whereas diabetic women with good glycemic control had lower vaspin levels than those with poor glycemic control in another study (50). Also, vaspin expression was shown to increase from overweight to obesity (51). In obese children, serum vaspin levels are positively correlated with TG, fasting insulin, and homeostatic model assessment of insulin resistance (HOMA-IR) (52). The body fat percentage was found to be the strongest predictor of visceral vaspin, and insulin sensitivity seems to be the strongest determinant of subcutaneous vaspin expression (51). On the other hand, no significant association between vaspin levels and glucose tolerance or insulin sensitivity has been reported in a cross-sectional study including 83 nondiabetic subjects (53). It is, therefore, still unclear whether the role of vaspin is causative or protective in the development of obesity and metabolic disorders.

There are few studies concerning the regulation of vaspin by thyroid hormones. Gonzalez *et al*. (54) investigated vaspin mRNA, glucose, and insulin levels in hyperthyroid, hypothyroid, and euthyroid rats. They showed that vaspin mRNA levels are significantly downregulated in hyperthyroid rats and significantly increased in hypothyroid rats compared with the euthyroid ones despite there being no change in glucose and insulin levels, suggesting that thyroid dysfunction may affect vaspin expression. Handisurya *et al*. (55) examined the relationship between TSH, vaspin, and leptin levels before and after weight loss by bariatric surgery. They reported a significant decrease in TSH levels in positive correlation with changes in serum vaspin levels. However, no definite conclusion can be drawn regarding whether vaspin causes the weight-loss-associated decrease of TSH levels, or whether changes in thyroid function or gastrointestinal peptides influenced serum vaspin levels. In a recent study, we have evaluated serum vaspin levels in hypothyroid patients before treatment and after establishment of euthyroidism (56). Vaspin levels were similar in euthyroid and hypothyroid subjects (subclinical and clinical hypothyroid), and no significant difference was observed in vaspin levels after normalization of thyroid hormones. Moreover, vaspin levels were not correlated with TSH. These data indicate that thyroid hormone status has no influence on serum vaspin levels in humans. More studies are needed to clarify the relationship between vaspin and thyroid hormones.

## Visfatin

Visfatin, previously defined as pre-B cell colony-enhancing factor, is a 52-kDa cytokine expressed and secreted by lymphocytes (57). Visfatin is also called nicotinamide phosphoribosyltransferase (NAMPT) because of its functional and biochemical homology with NAD biosynthesized from nicotinamide (58). Fukuhara *et al*. (59) used the term ‘visfatin’ for this protein due to its predominant production in the VAT. Visfatin has insulin-mimicking/-sensitizing effects. Visfatin binds to the insulin receptor at a site distinct from insulin and exerts hypoglycemic effect by reducing glucose release from hepatocytes and stimulating glucose utilization in the peripheral tissues (60). Increased serum visfatin concentrations have been observed in type 2 diabetes, obesity, polycystic ovary syndrome, and nonalcoholic fatty liver disease. Weight loss after an exercise program and bariatric surgery results in a decrease in visfatin levels along with improvement in insulin sensitivity, indicating a compensatory mechanism in response to hyperglycemia associated with insulin resistance. Moreover, visfatin plays a role as an important mediator of inflammation, inducing the secretion of various proinflamatory and anti-inflammatory cytokines (61).

The relationship between visfatin and thyroid hormones was examined in a few studies. Experimental studies have revealed controversial results indicating that T_3_ could accelerate adipocyte differentiation with the elevation of visfatin levels (62), whereas MacLaren *et al*. (63) have reported downregulation of visfatin mRNA expression by T_3_ in 3T3-L1 adipocytes.

Chu *et al*. (64) evaluated the change in visfatin, C-reactive protein (CRP) concentration, and insulin sensitivity in 19 patients with hyperthyroidism due to Graves' disease and 19 age- and sex-matched controls. The hyperthyroid group had significantly higher plasma visfatin levels than controls, and visfatin levels significantly decreased after treatment. Visfatin, glucose, insulin, and HOMA-IR were values positively correlated with T_3_ and fT_4_ levels, but no significant association was found among visfatin levels and insulin and HOMA-IR values. The data suggest that the higher concentration of visfatin in the hyperthyroid patients may be related to a state of visfatin resistance, and that insulin resistance in hyperthyroidism is not associated with visfatin.

Caixas *et al*. (65) evaluated plasma visfatin, IL6, CRP, ADP, and insulin sensitivity parameters in 24 hyperthyroid and 27 hypothyroid patients before and after treatment in comparison with 45 euthyroid subjects. Hyperthyroid patients had significantly increased insulin resistance, IL6, and visfatin levels compared with controls. Visfatin levels increased after treatment, while IL6 levels and HOMA-IR decreased. CRP and ADP levels were similar in the hyperthyroid and control groups. Hypothyroid patients had higher visfatin levels compared with healthy subjects, which further increased after treatment without changes in anthropometric and insulin resistance parameters. No significant correlations between visfatin and any other parameters were found. It is suggested that visfatin might play a role in the recovery period independent of anthropometric, inflammatory, or insulin resistance parameters. Ozkaya *et al*. (66) studied the serum visfatin levels in 56 Hashimoto's thyroiditis patients with hypothyroidism, 56 Graves' patients with hyperthyroidism, and 56 euthyroid healthy subjects before and after treatment. Hyperthyroid patients had significantly lower visfatin levels compared with the hypothyroid group and controls. Plasma visfatin level decreased significantly after treatment in the hypothyroid group, whereas it increased significantly after treatment in the hyperthyroid group. A significant positive correlation between visfatin and TSH levels and a significant negative correlation between visfatin levels and fT_3_ and fT_4_ values were observed.

Han *et al*. (67) studied the regulation of visfatin by thyroid hormones *in vivo* and *in vitro*. The *in vivo* experiment included 57 patients with thyroid dysfunction and 29 euthyroid subjects and an animal model (24 Wistar rats). The *in vitro* experiment included 3T3-L1 cells and visfatin mRNA expression in the visceral fat and liver of rats under different T_3_ concentrations. Clinical subjects and animal models had elevated plasma visfatin concentrations in both hypo- and hyperthyroid groups compared with controls. For animal models, visfatin mRNA expression was found to be increased in only visceral fat but not liver in hypo- and hyperthyroid groups compared with controls, along with a positive correlation with plasma visfatin levels. T_3_ induced a remarkable increase in visfatin mRNA expression in 3T3-L1 cells at low concentrations followed by a sharp decrease at higher concentrations. The data indicate that thyroid dysfunction is associated with elevated visfatin levels, possibly due to an increase in visfatin mRNA expression in visceral fat, and that T_3_ caused a nonlinear regulation of visfatin mRNA expression.

Controversial results are reported concerning the role of thyroid hormones in the regulation of visfatin, with regard to the studies mentioned earlier. Such discrepancies might be explained by differences in ethnic or methodological factors or heterogeneity of thyroid dysfunction.

High levels of visfatin were observed in other autoimmune diseases such as rheumatoid arthritis or inflammatory bowel disease (61). Autoimmune thyroid dysfunction may be closely associated with fluctuations in visfatin levels. Proinflammatory cytokines such as IL6 and tumor necrosis factor α (TNFα) are increased in patients with thyroid dysfunction (68, 69), and IL6 has the ability to induce the expression of visfatin *in vitro* (70). Therefore, it is likely that visfatin release from adipose tissue may be affected directly or indirectly via proinflammatory cytokines implicating them in thyroid dysfunction. Visfatin is also expressed in the skeletal muscle (57). Both skeletal visfatin expression and plasma levels increase together with muscle mass growth, making it act as a myokine (71). Hyperthyroid patients usually lose lean body mass, which is supposed to be accompanied by decreased visfatin secretion. However, increased visfatin levels were also observed in hyperthyroidism, while decreased levels were reported in hypothyroid patients. This might be due to the compensatory increase in visfatin levels against the high metabolic rate accelerating the breakdown of fat in hyperthyroidism.

In conclusion, the pathophysiological role of thyroid hormones in the regulation of ADP, vaspin, and visfatin is still unclear. Changes in adipokine secretion with thyroid dysfunction may represent adaptive mechanisms to the decrease or increase in basal energy expenditure and in energy substrate requirements in thyroid dysfunction. Cytokine network imbalances may be involved in the interactions between thyroid hormones and cytokines. Moreover, hyper- and hypothyroidism could affect the clearance of these cytokines. Additional studies, particularly studying the interactions among the novel genes in the adipose tissue, adipocytokines and the thyroid, will generate further insights into the endocrine function of adipose tissue.

## Figures and Tables

**Table 1:** Summary of previous studies of adiponectin and thyroid hormone association.

**Study**	***n***	**Results**	**Correlations**
Positive association			
(15)	68 healthy subjects		Positive ADP vs fT_4_
(24)	69 hyperthyroid patients	↑ ADP in hyperthyroid patients	Positive ADP vs fT_4_
		↓ ADP after establishment of hypothyroidism (*n*: 32 patients)	Positive ADP vs fT_3_
(25)	32 hyperthyroid Graves' patients	↑ ADP levels in hyperthyroid than in euthyroid patients and controls	Positive ADP vs fT_4_
	32 euthyroid Graves' patients	Positive ADP vs fT_3_
	30 controls		Positive ADP vs TRAb (TRAb – the best predictor of ADP levels)
(26)	46 hyperthyroid patients	↑ ADP levels in hyperthyroid patients than controls	Positive ADP vs fT_4_
	23 hypothyroid patients	↔ ADP levels in hypothyroid patients	Positive ADP vs fT_3_
	30 controls		Positive ADP vs insulin and HOMA-IR
			Negative ADP vs total cholesterol, BMI, and triglyceride
(27)	39 hyperthyroid patients	No significant difference in ADP levels between the groups	Positive ADP vs fT_4_
23 controls	↔ ADP after normalization of thyroid status	Positive ADP vs BMI and WC
(28)	53 hypothyroid patients	↓ ADP levels in hypothyroid patients and after normalization of thyroid status	Positive ADP vs WC and weight
	30 controls
(29)	76 hyperthyroid Graves' patients (26 without GO and 50 with GO)	↑ ADP levels in hyperthyroid patients than controls	Positive ADP vs fT_4_
	30 controls	↔ ADP between patients with GO vs without GO	Positive ADP vs fT_3_
		↔ ADP between patients with active GO vs inactive GO	Positive ADP vs TRAb
(30)	120 hyperthyroid patients	↓ ADP levels after normalization of thyroid status	Positive ADP vs fT_4_
	Negative ADP vs BMI (BMI – the best predictor of ADP levels)
(31)	28 thyroid carcinoma patients	↔ ADP 4 weeks after thyroid hormone withdrawal	Positive ADP vs fT_4_
(32)	234 euthyroid prepubertal children		Positive ADP vs fT_4_
(33)	321 healthy euthyroid pregnant women (24–28 weeks gestation)	Positive HMW ADP vs fT_4_
			Negative HMW ADP vs fT_3_:fT_4_ ratio
No association			
(34)	20 hyperthyroid patients	No significant difference in ADP levels among the groups	↔ ADP vs HOMA-IR, glucose, and insulin
	20 hypothyroid patients	↔ ADP in both hypo/hyperthyroid groups, after normalization of thyroid status	
	20 euthyroid subjects		
(35)	15 hyperthyroid patients	No significant difference in ADP levels among the groups	
	15 hypothyroid patients
	15 controls		
(36)	22 women with differentiated thyroid carcinoma	↔ ADP with thyroid hormone withdrawal	
(37)	67 hypothyroid patients	No significant difference in ADP levels among the groups	Positive ADP vs HDL
	56 hyperthyroid patients	Negative ADP vs BMI
	52 controls		
(38)	98 euthyroid postmenopausal women with Hashimoto's thyroiditis	No significant difference in ADP levels between the groups	↔ ADP vs TSH, fT_4_, and TPO Abs
	105 postmenopausal controls		
(39)	19 hyperthyroid Graves' disease	No significant difference in ADP levels between the groups	
	19 controls	↔ ADP after normalization of thyroid status	
(40)	98 obese euthyroid women	No significant difference in ADP levels between the groups	↔ ADP vs thyroid volume
	31 non-obese euthyroid women	↔ ADP after weight loss
(41)	30 premenopausal euthyroid women with nodular goiter	↔ ADP short-term after thyroidectomy-induced hypothyroidism despite significant ↑ in BMI and serum lipid levels	↔ ADP vs thyroid hormones
(42)	43 subclinical hypothyroid patients	No significant difference in ADP levels between the groups	
	53 controls	↑ ADP after normalization of thyroid status	

↑, Increase; ↓, decrease; ↔, no change; ADP, adiponectin; fT_4_, free thyroxine; fT_3_, free triiodothyronine; TPO Abs, thyroid peroxidase autoantibody; TRAb, TSH-R antibodies; WC, waist circumference; HMW, high molecular weight; GO, Graves' ophthalmopathy.
